# Evaluation of the Allergenicity Potential of TcPR-10 Protein from *Theobroma cacao*

**DOI:** 10.1371/journal.pone.0037969

**Published:** 2012-06-29

**Authors:** Sara Pereira Menezes, Jane Lima dos Santos, Thyago Hermylly Santana Cardoso, Carlos Priminho Pirovani, Fabienne Micheli, Fátima Soares Motta Noronha, Andréa Catão Alves, Ana Maria Caetano Faria, Abelmon da Silva Gesteira

**Affiliations:** 1 UESC, Centro de Biotecnologia e Genética, Ilhéus, Bahia, Brasil; 2 UESC, Centro de Microscopia Eletrônica, Ilhéus, Bahia, Brasil; 3 CIRAD, UMAR AGAP, Montpellier, France; 4 UFMG, Instituto de Ciências Biológicas, Pampulha, Belo Horizonte, Minas Gerais, Brasil; 5 Embrapa Mandioca e Fruticultura, Cruz das Almas, Bahia, Brasil; Naval Research Laboratory, United States of America

## Abstract

**Background:**

The pathogenesis related protein PR10 (TcPR-10), obtained from the *Theobroma cacao*-*Moniliophthora perniciosa* interaction library, presents antifungal activity against *M. perniciosa* and acts *in vitro* as a ribonuclease. However, despite its biotechnological potential, the TcPR-10 has the P-loop motif similar to those of some allergenic proteins such as Bet v 1 (*Betula verrucosa*) and Pru av 1 (*Prunus avium*). The insertion of mutations in this motif can produce proteins with reduced allergenic power. The objective of the present work was to evaluate the allergenic potential of the wild type and mutant recombinant TcPR-10 using bioinformatics tools and immunological assays.

**Methodology/Principal Findings:**

Mutant substitutions (T10P, I30V, H45S) were inserted in the *TcPR-10* gene by site-directed mutagenesis, cloned into pET28a and expressed in *Escherichia coli* BL21(DE3) cells. Changes in molecular surface caused by the mutant substitutions was evaluated by comparative protein modeling using the three-dimensional structure of the major cherry allergen, Pru av 1 as a template. The immunological assays were carried out in 8–12 week old female BALB/c mice. The mice were sensitized with the proteins (wild type and mutants) via subcutaneous and challenged intranasal for induction of allergic airway inflammation.

**Conclusions/Significance:**

We showed that the wild TcPR-10 protein has allergenic potential, whereas the insertion of mutations produced proteins with reduced capacity of IgE production and cellular infiltration in the lungs. On the other hand, *in vitro* assays show that the TcPR-10 mutants still present antifungal and ribonuclease activity against *M. perniciosa* RNA. In conclusion, the mutant proteins present less allergenic potential than the wild TcPR-10, without the loss of interesting biotechnological properties.

## Introduction

The development of genetically modified organisms (GMOs) through molecular engineering techniques is an alternative to plant genetic improvement programs for the purpose of promoting resistance against pathogens, herbicides or environmental stresses [1], [2]. Among the genes that can be potentially used in the genetic transformation of plants so as to improve resistance against diseases, those coding for pathogenesis-related proteins stand out (PR) [3], [4], [5], [6], [7]. According to the structural, enzymatic or biological properties, PR proteins are classified into 17 families, whereas PR 1, 2, 3, 4, 5, 8, 10 and 14 are reported to contain proteins with homology to pollen or food allergens; this fact limits the biotechnological application of these genes [8], [9], [10], [11]. Among the allergenic proteins classified as PR-10, the Bet v 1 isolated from *Betula verrucosa* is the main allergen present in pollen grains [12], [13]. Food allergens such as Pru p 1 from pear (*Prunus persica*) [14], Mal d 1 from apple (*Malus domestica*) [15], Pru av 1 from cherry (*P.avium*) [16], [17] and Dau c 1 in carrot (*Daucus carota*) [18] are also reported as part of the PR-10 family.

The PR-10 family is characterized by the presence of a highly conserved region called P-loop motif, which is usually associated with ribonuclease activity in some members of this family [19]. Yet, the presence of this domain is also associated with the allergenicity of pollen grains [20], [21]. The P-loop motif present in some allergenic proteins such as Mal d 1 (*Malus domestica*) [22], Bet v 1 (*Betula verrucosa*) [12] and Api g 1 (*Apium graveolens*) [23] is also conserved in the *TcPR-10* gene identified in a cDNA library observed in the interaction between *Theobroma cacao* and *Moniliophthora perniciosa*.

The TcPR-10 protein has a promising biotechnological potential to act as a ribonuclease and presents antifungal activity against *M. perniciosa*, the causal agent of witches’ broom disease, which is one of the most devastating diseases of cocoa plants [24]. The overexpression of the *TcPR10* gene may open new possibilities for cocoa breeding. However, the development of genetically modified organisms (GMOs) requires the discrimination of allergenic and non-allergenic recombinant proteins and a prediction of the potential cross-linking activity of the proteins of the immune system [25], [26]. The assessment of allergenicity potential is a major procedure used to ensure the biosafety of GMOs [27]. Thus, this study has aimed to assess the allergenicity potential of the antifungal protein TcPR-10 using bioinformatic tools and immunological assays, and develop and test a mutant strain with little or no allergenic ability, but that maintains ribonuclease and antifungal activities.

## Results

### Identification of the Allergenicity Potential of TcPR-10 through Bioinformatics Analysis

**Figure 1:**
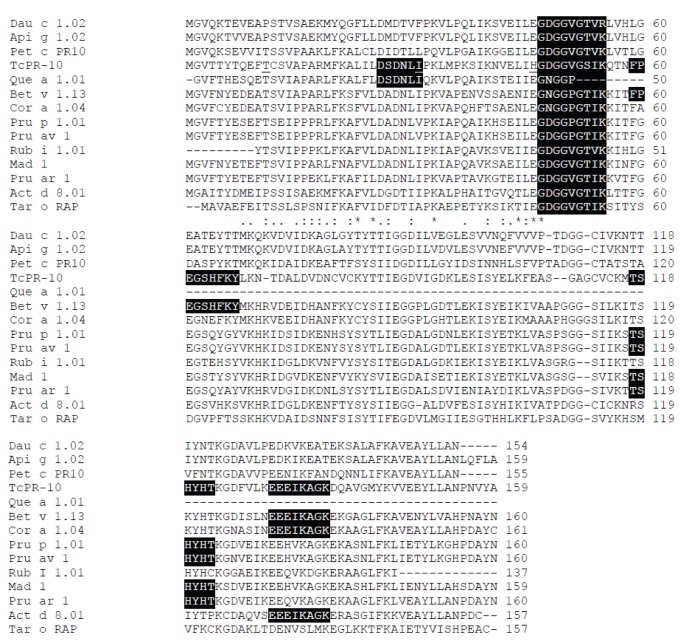
Amino acid sequence alignment of TcPR-10 with the allergens from the SDAP database ( http://align.genome.jp/sit-bin/clustalw **).** The three point mutations for TcPR-10 (T10P, I30V, H45S) are marked in sequence alignment and P-loop was underlined. The identical, highly conserved, and conserved amino acids among the sequences are denoted with (*), (:), and (.), respectively. Matching regions of contiguous amino acids are highlighted in black.

The assessment of the allergenicity potential of the TcPR-10 protein by sequence comparison analysis with sequences of allergens from the SDAP (Structural Database of Allergenic Proteins) [28], [29] database revealed similarity to 13 different groups of allergens (Table 1). The TcPR-10 sequence shows stretches of 6 continuous and identical amino acids with food allergens like Rub i (red raspberry), Dau c 1.01 (carrot), Act d 8 (kiwi fruit), Api g 1 (celery), Mad 1 (apple), Pru ar 1 (apricot), Cor a 1.04 (hazelnut), Pru p 1 (peach), Pru av 1 (sweet cherry), and also pollen allergens such as Que a 1 (white oak) and Bet v 1 (white birch). Based on the sequence of continuous amino acids, one should note that the *TcPR-10* gene showed similarity to allergenic proteins especially in the region rich in glycine (P-loop motif 47GDGGVGSIK55) (Figure 1). Despite the fact that the P-loop motif of the TcPR-10 protein is not identical to Pru p 1, Pru av 1, Bet v 1, Que a 1 and Cor a 1, these proteins also have a P-loop and there are amino acid sequence variations among glycine residues. At position 48, the Bet v 1, Que a 1 and Cor a 1 sequences contain asparagine residues, whereas the TcPR-10 protein contains aspartic acid residues. At position 51, the Pru p 1, Pru av 1, Bet v 1, Que a 1 and Cor a 1 proteins show a proline residue, whereas TcPR-10 shows a valine residue. In addition to the P-loop domain, theTcPR-10 protein sequence shows the common 129EEEIKAGK136 region with Bet v 1, Cor a 1 and Act d 8, the common 116TSHYHT121 region with Mal d 1, Pru ar 1, Pru p 1.0101 and Pru av 1, the 26DSDNLI31 region only with Que a 1, and 59FPEGSHFKY67, only with Bet v 1 (Figure 1).

**Table 1:** Identification of potential allergenic of TcPR-10 protein in the SDAP^1^ allergenic proteins database.

N°	Allergen	Species	Description	Sequence in SDAP^1^	Sequence Length	Matching region of contiguous amino acids	E-value	PD^2^	Start-End of the alignment withTcPr-10	Identity^3^
1	Que a 1.0101	*Quercus Alba*	Major oak pollen allergen	P85126	50	25DSDNLI30	1.5e^−07^	6.13	2–51	17.72
2	Rub i 1.0101	*Rubus idaeus*	Putative allergen	Q0Z8U9	137	38GDGGVG43	3.8e^−25^	13.70	10–146	43.04
3	Dau c 1.0201	*Daucus carota*	Food allergen	AAL76932	154	47GDGGVG52	8.1e^−18^	13.66	1–154	35.44
4	Pet c PR10	*Petroselinum crispum*	Pathogenesis-related protein 1	CAA67246	155	47GDGGVG52	9.7e^−21^	13.94	1–155	39.87
5	Act d 8.0101	*Actinidia deliciosa*	Food allergen andBet v 1 related allergen	CAM31909	157	47GDGGVG52131EEEIKAGK139	4.3e^−24^	12.13	1–157	48.73
6	Tar o RAP	*Taraxacum officinale*	Root allergen-and pathogenesis-related protein	AAB92255	157	47GDGGVG52	9.3e^−18^	16.21	1–157	35.44
7	Api g 1.0201	*Apium graveolens*	Food allergen	P92918	159	47GDGGVG52	3.9e^−21^	13.62	1–159	38.61
8	Mal d 1	*Malus domestica*	Food allergen	CAA96534	159	47GDGGVG52117TSHYHT122	1.5e^−30^	12.67	1–159	53.16
				CAA96535	160	47GDGGVG52	1.7e^−28^	13.39	1–158	54.43
				CAA96536	160	47GDGGVG52	1.9e^−28^	13.34	1–158	53.80
				CAA96537	160	47GDGGVG52	1.9e^−28^	13.22	1–158	54.43
9	Pru ar 1	*Prunus armeniaca*	Food allergen	O50001	160	47GDGGVG52118TSHYHT123	7.4e^−31^	13.12	1–158	53.80
10	Pru p 1.0101	*Prunus persica*	Food allergen and Pathogenesis related protein PR10	Q2I6V8	160	118TSHYHT123	2.1e^−29^	13.38	1–158	51.90
11	Pru av 1	*Prunus avium*	Food allergen	O24248		118TSHYHT123	5.5e^−29^	13.30	1–158	51.27
12	Bet v 1.1301	*Betula verrucosa*	Major pollen allergen	CAA54696	160	59FPEGSHFK67131EEEIKAGK138	3.9e^−30^	13.46	1–158	54.43
13	Cor a 1.0401	*Corylus avellana*	Food allergenand Bet v 1-related majorhazelnut allergen	AAD48405	161	132EEEIKAGK139	3.3e^−26^	14.31	1–161	49.37
	Cor a 1.0404	*Corylus avellana*		AAG40331	161	132EEEIKAGK139	5.0e^−26^	14.25	1–161	48.73
	Cor a 1.0403	*Corylus avellana*		AAG40330	161	132EEEIKAGK139	2.2e^−26^	14.34	1–161	50.00
	Cor a 1.0402	*Corylus avellana*		AAG40329	161	132EEEIKAGK139	1.4e^−26^	13.56	1–161	50.00

1 Structural database of allergenic proteins; ^2^Property distance index; ^3^Identity for two sequences.

Based on the identity parameter greater than 35% in a window of 80 amino acids, the TcPR-10 sequence has an excess of 50% homology with the Mad 1, Pru ar 1, Pru p 1.0101, Bet v 1 Cor a 1.0403 and Cor a 1.0402 sequences. The percentage similarity for these six allergen sequences is consistent with the PD value under 14, thus indicating similarity between the physicochemical properties of such allergens and TcPR-10. It can be noted that the lowest and therefore most significant overall similarity index values (E-value) are also observed for these allergen sequences. *E-value* and *PD* indexes indicate that TcPR-10 and *PD* show similarity to pollen and food allergens (Table 1).

### Changes in the Structure of the Hydrophobic Cavity and the Surface of TcPR-10

**Figure 2:**
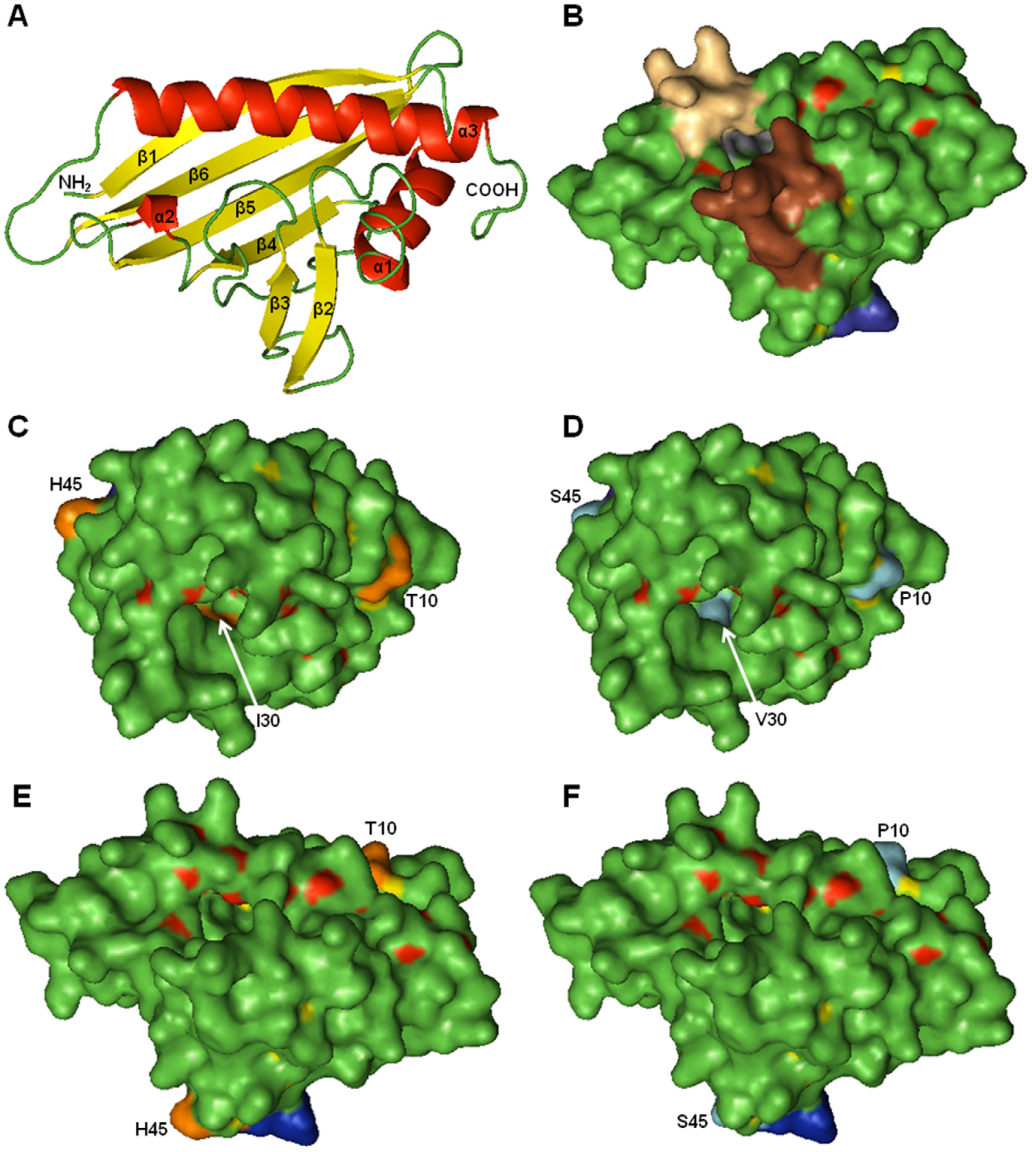
Three-dimensional structure of TcPR-10 obtained by homology modeling with Pru av1 (Protein Data Bank, 1e09_A) as template using SWISS-MODEL. A. The secondary structure elements are colored: alpha-helices in red, anti-parallel beta-sheets in yellow and P-loops in green. **B.** Molecular surface of TcPR-10 wild with matching regions of contiguous amino acids: 47GDGGVG52 in blue; 59FPEGSHFKY67 in brown; 116TSHYHT121 in gray; 129EEEIKAGK136 in peach. **C** e **E** Molecular surface of TcPR-10 wild type with amino acids for mutations highlighted in orangen (Thr10, Ile30, His45). **D e F.** TcPR-10 mutant type with point mutations in blue (Pro10, Val30, Ser45).

In order to select sites for insertion of mutations that reduce or eliminate the possible allergenic potential of TcPR-10 shown by bioinformatics analysis using SDAP, molecular modeling has been employed so as to predict changes and determine the implications that they may cause in the tridimensional structure of TcPR-10. The templates were identified by PSI-Blast [30] analyses against protein data banking-PDB [31]. The sequences of wild and mutant-type TcPR-10 showed 51% identity in relation to the templates Pru av 1 (pdb: 1e09_A) and RMSD of 0.345 and 0.351, respectively (Table 2). Identity above 50% and an E-value below 4e^−43^ indicate that the crystal structure of Pru av 1 is a good model to be used as a template. The value of RMSD indicates that there was little difference between models and template structures. The models of wild and mutant-type TcPR-10 showed three alpha-helices: α1 (16–26); α2 (89–91); α3 (129–152); six stranded anti-parallel beta-sheets (β1-β6): β1 (3–12); β2 (40–46); β3 (54–58); β4 (80–85); β5 (96–106); β6 (111–121); and 9 loop L1 (13–15); L2 (27–39); L3 (47–53); L4 (59–79); L5 (86–88); L6 (92–95); L7 (107–110); L8 (122–128); L9 (153–158) (Figure 2A).

**Table 2:** Template obtained by PSI-Blast algorithm for modeling of proteins structures TcPR-10 wild and mutant.

	Template	Identify %	E-value	Organism	RMSD(Å)	Reference
TcPR-10 wild	1e09.pdb	51	4e^−43^	*Prunus avium*	0.345	Neudecker et al., 2001
TcPR-10 Mutant	1e09.pdb	51	2e^−42^	*Prunus avium*	0.351	Neudecker et al., 2001

The stereochemical parameters of the model protein structural wild type and mutant were analyzed using the Procheck (Laskowski et al., 2005) and Anolea softwares (Melo; Feytmans, 1998). The Ramachandran plot showed that 76,1% and 76,6% of residues in most favored regions for wild and mutant-type TcPR-10, respectively. 21,7% and 21,2% of residues in additional allowed regions and only 2,2% and 2,2% residues in disallowed regions for wild and mutant-type TcPR-10, respectively. Analysis of the stereochemical properties with Procheck 3.4 confirms good stereochemical quality of the structure ensemble as mirrored by the fact that more than 97% of the amino acid residues are located in the most favoured regions of the Ramachandran plot.

### Cloning and Expression of TcPR-10

**Figure 3:**
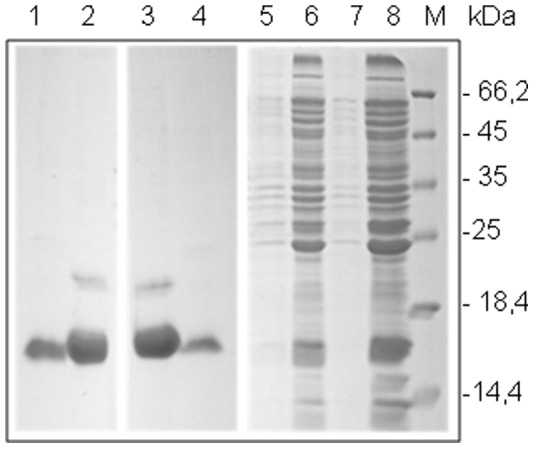
SDS-PAGE (15%) analysis of recombinants TcPR-10 Wild Types (wt) and Mutant (mut) proteins expressed in *E. coli* BL21(DE3). **1**– Soluble fraction TcPR-10 mut; **2**– Insoluble fraction TcPR10 mut; **3**– Soluble fraction TcPR-10 wt; **4**– Insoluble fraction TcPR10 wt; **5** - pET28a-TcPR-10 mut without induction; **6** - pET28a-TcPR-10 mut after induction; **7** - pET28a-TcPR-10 wt without induction; **8** - pET28a-TcPR-10 mut 3 h after induction; **M**- Protein molecular weight markers.

The recombinant plasmid pET28a containing the TcPR-10 insert with the T10P, I30V and H45S substitutions was cloned and expressed in *E. coli* BL21 (DE3) under the control of the T7 promoter. The three points of substitutions T10P, I30V and H45S-10 inserted into TcPR-10 were marked underlined in the alignment (Figure 1). The heterologous expression of the protein was confirmed in 15% SDS-PAGE gel stained with Coomassie Brilliant Blue and the expression of peptides of an approximate molecular mass of 19KDa fused to the histidine residues, was observed (His-Tag). The purification of the wild and mutant-type TcPR-10 was confirmed by visualization of single bands on SDS-PAGE gel (Figure 3).

### Ribonuclease and Antifungal Activity of the mutanttcpr-10

**Figure 4:**
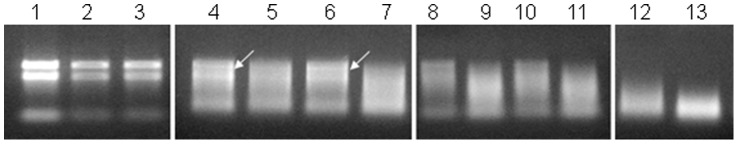
Ribonuclease activity of recombinant TcPR-10 Wild Types (wt) and Mutant visualized in 1% agarose gel. 1 µg RNA from *M. perniciosa* was incubated with 1 µg of recombinats proteins at 25°C at different times. Lane **1.** RNA without protein; Lane **2.** RNA with boiled TcPR10 mut 2 h incubation; Lane **3.** RNA with boiled TcPR10 wt 2 h incubation; Lane **4, 6, 8, 10** and **12** RNA incubated with TcPR10 mut by 10 min, 20 min, 1 h, 2 h and 3 h, respectively; Lane **5, 7, 9, 11** and **13** RNA incubated with TcPR10 wt by 10 min 20 min, 1 h, 2 h and 3 h, respectively. Arrows indicate RNA bands without degradation.

The verification of possible changes in the catalytic function of the TcPR-10 protein due to the insertion of nucleotide substitutions was performed in vitro, using RNA from *M. perniciosa*. Incubation of the mutant TcPR-10 protein with RNA from *M. perniciosa* at 25°C at different times shows that, although there has been a slight decrease in the degradation rate, the insertions have not altered the activity of the ribonuclease protein. After 10 minutes of incubation with the *TcPR-10* mutant gene, the RNA was not completely degraded, as observed for the wild-type TcPR-10 (Figure 4, line 4). After one hour, the RNA bands incubated with the mutant TcPR-10 were still present, but after 3 hours, the RNA from *M. perniciosa* was observed to be totally degraded both in the wild-type and the mutant protein (Figure 4, row 6).

**Figure 5:**
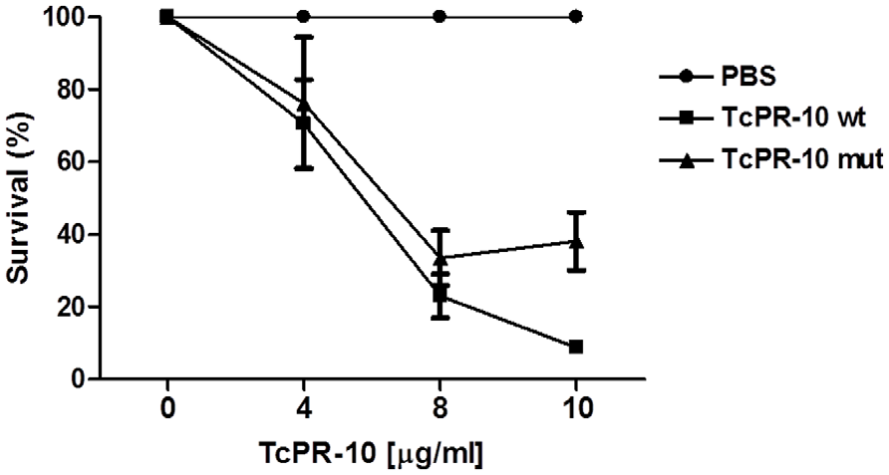
Survival of *M. perniciosa* dikaryotic broken hyphae incubated with different TcPR-10 wild and mutant type protein concentrations (4, 8 and 10 µg/ml).

The *in vitro* effect of the TcPR-10 mutant protein in the survival of *M. perniciosa* shows that changes do not inhibit the antifungal activity of the protein. The survival of the fungus decreases as the concentration of mutant TcPR-10 increases, showing the same profile observed for the wild protein (Figure 5). At a concentration of 8 mg/mL, the wild TcPR-10 protein shows a 73% inhibition rate of growth for the fungus, and 61% for the mutant TcPR-10 protein, with no statistical difference between values (p>0.05;Tukey’s test). Despite the fact that the wild-type protein shows a higher percentage of inhibition as compared with the mutant protein especially when the concentration increases to 10 µg/mL, reducing the growth of the fungus by 91% - the mutant protein only reduces 56% - these values have not statistically differed (p>0.05, Tukey’s test).

### Immunological Response to *TcPR-10*

**Figure 6:**
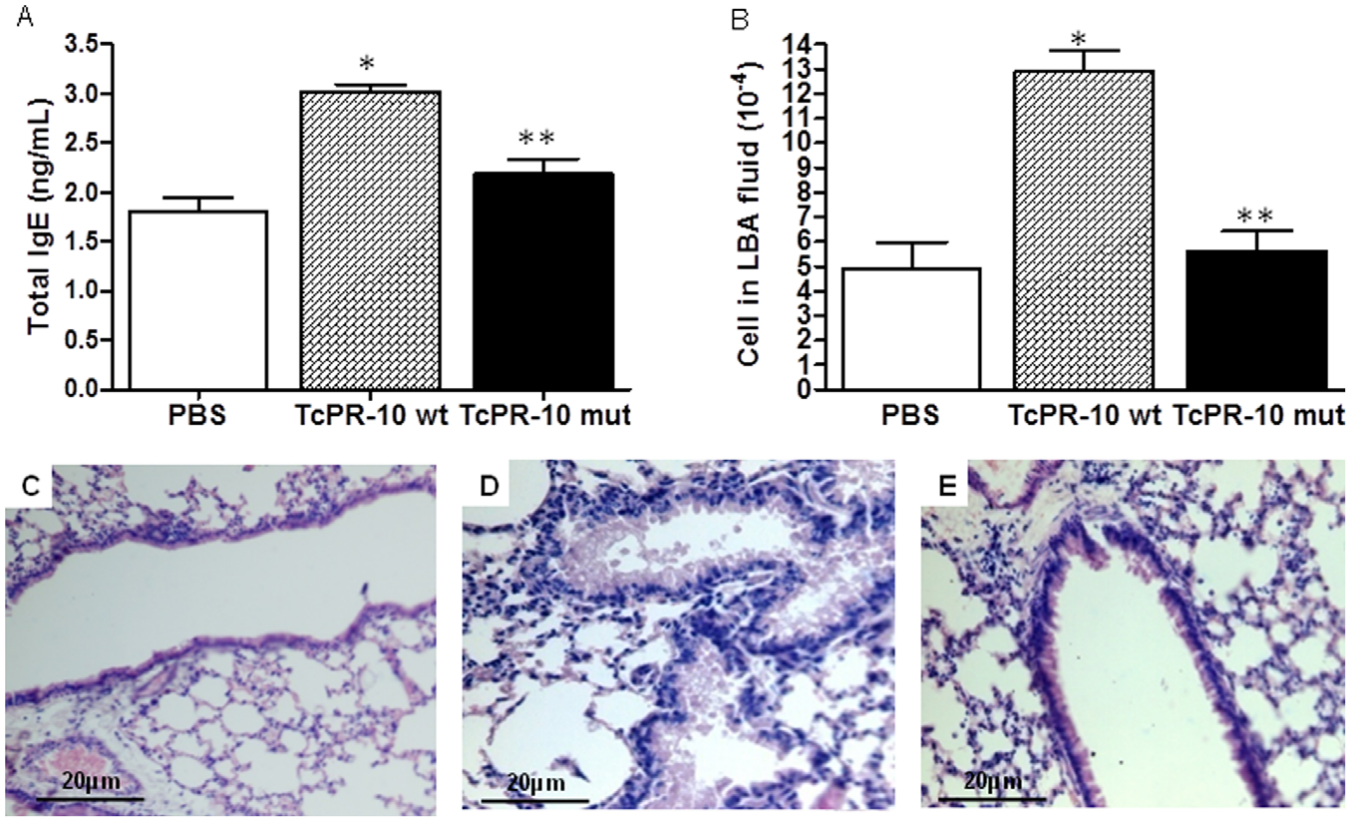
Quantification of polyclonal IgE, BAL total cell count and histological illustration of the lung of BALB/c mice. **A.** Quantification of polyclonal IgE antibody levels in serum of BALB/c mice. **B.** Cell counting in BAL fluid. The set average values *per se* quantification of antibodies and showed normal (p<0,05; Shapiro Wilk Test) using the comparison test of means the parametric Tukey Test (α = 0,05). *Significantly high values compared to control. **Significantly reduced values compared to TcPR-10 wt. Horizontal bars represent the mean value of each group. C. Lung were removed twenty-four hours after the last challenge. Lung tissue was fixed, embedded, cut into slices and stained with hematoxylin e eosin (H&E) solution. **C:** Sections from control; **D:** wild TcPR-10; **E:** mutant TcPR-10.

The production of immunoglobulin E (IgE) stimulated by an allergen usually triggers the typical symptoms of hypersensitivity reactions. In order to evaluate the effect of wild and mutant TcPR-10 on airway inflammation, the IgE levels in serum and infiltration of inflammatory leukocytes in the lung have been examined. IgE levels in serum of animals challenged with the wild TcPR-10 protein has increased by 40% (M: 3.02; SD: +0.16) as compared to control animals (M: 1.8; SD: +0.32) (p<0.001; Tukey’s test) (Figure 6A). On the other hand, those treated with mutant TcPR-10 have only increased by 17% (M: 2.18; SD: +0.34) (p>0.05; Tukey’s Test) as compared to the control (Figure 6A). The IgE levels in animals treated with mutant protein is about 27% lower as compared with animals that received the wild-type TcPR-10, accordingly supporting the hypothesis that the insertion of mutations in TcPR-10 can reduce the allergenicability of this protein. The infiltration of inflammatory leukocytes was investigated by the following parameters: (i) total cell count in the BAL bronchoalveolar lavage fluid and (ii) analysis of histological sections. The total number of cells in BAL from animals treated with wild TcPR-10 protein (M: 12.95×104; D:+1.82×104) was higher than that present in the mutant and control groups, hence indicating increases of 62% (4.9×104+1.08×104) and 57% (M: 5.62×104; D: +1.81×104) (p<0.001, Tukey’s test), respectively (Figure 6B).When comparing the mutant TcPR-10 protein with the control group, there was no significant difference but a 12.81% increase (p>0.05; Tukey’s test). The histological analysis of lungs of mice treated with the wild-type TcPR-10 protein shows discrete cellular infiltrate in the peribronchiolar region and mild desquamation of the epithelium, with presence of mucus in the lumen of the bronchi (Figure 6D). These changes were not seen in the lungs of mice treated with the mutant protein (Figure 4E) and neither in the control animals (Figure 6C).

## Discussion

The pathogenesis-related proteins (PR) involved in plant defense response against pathogens - including PR class 10 - are reported to be constitutively expressed in pollen, fruits and vegetables and may cause allergic reactions in humans [32], [33], [34]. The ribonuclease and antifungal activities of the heterologous protein TcPR-10 makes it important to the defense of *T. cacao* against *M. perniciosa*. This fact suggests that the *TcPR-10* gene can be used to increase the resistance of plants to pathogens [24] and is therefore subject of considerable biotechnological interest. Yet, despite the importance of biotechnology, the allergenicity potential of TcPR-10 had not been previously reported.

The identification of the allergenicity potential of proteins is usually conducted using bioinformatics tools and immunological assays [26], [35], [36]. In conjunction with the Food and Agriculture Organization of the United Nations [37], the World Health Organization has recommended two criteria using bioinformatics analysis to identify the allergenic potential of proteins, both based on sequence alignments: one that indicates identity greater than 35% in a window of 80 amino acids and another with 6 continuous amino acids between the sequence examined and those available in the databases and already reported to induce IgE production [38], [39], [40], [41].

According to these parameters, the TcPR10 sequence showed similarity with thirteen allergenic sequences, and the region rich in glycine (P-loop motif 47GXGGXGXXK55) was highly conserved in all sequences (Table 1). Among the allergens that showed similarity with TcPR-10, Dau c 1, Pet c PR10, Tar o RAP, Api g 1, Mal d 1, Pruar 1, Pruav 1, Bet v 1 Cora 1 are reported as belonging to the family of pathogenesis-related proteins 10 - Bet v 1 (PF00407) [26]. The analysis of X-ray diffraction of protein Bet v 1 has identified several regions responsible for intermolecular contacts with the monoclonal Fab fragment of the IgG1 molecule (BV16), the region 42ENIEGNGGPGT52 (corresponding to the P-loop) identified as the main binding epitope [20]. Other sites of contact have been identified: R70, D72, H76, I86, E87 and K97 [20]. The TcPR-10 protein has the same amino acid residues at positions 72, 86 and 87, located near the conserved region 59FPEGSHFKY67, thus suggesting that these regions may act as binding sites for IgE. The crystalized structure of the Bet v 1-BV16 Fab complex demonstrated that the epitope formed by these amino acids, is clearly conformational [21], however, it is interesting to notice that the P-loop region is also a sequential motif [21]. The P-loop has been suggested as an important area of the epitope also in the ligation of IgE to Bet v 1. Mutations in the E45 residue, located in the middle of the BV16 epitope, was defined as the dominant epitope in the ligation of IgE in the serum of patients allergic to birch [21]. The residue of the amino acid 45, although not conserved in TcPR-10 (His-45), also is localized structurally in the superficial area which covers the location of the P-loop. In Pru av 1, the mutation in the E45W amino acid, also located in the P-loop region, does not alter the tertiary structure of the protein, but alters the biophysical properties of the lateral chain, and also reducing significantly the capacity of ligation to IgE for Pru av a in the serum of patients allergic to cherry [42]. In Bet v 1, other residues such as Asn-28, Lys-32 and Pro-108, also had a significant contribution as to the ligation to IgE [21]. Residue 28 is also considered in Pru av 1 as a second region of the IgE epitope [42].

Conserved areas of the molecular surface are listed as probable IgE-binding epitopes [16]. The modeling of TcPR-10 protein shows that the conserved regions with allergens in SDAP 59FPEGSHFKY67, 116TSHYHT121 and 129EEEIKAGK136 are located near the hydrophobic cavity (Figure 2B); it is relevant to speculate whether these conserved areas indeed represent the IgE binding epitopes. The internal cavity present in the PR-10 protein is reported as a binding site or reservoir for hydrophobic ligands in the aqueous environment of the cell [3], [12], [43]. The location of these conserved regions close to the cavity enables one to underscore the importance of the cavity for the biological function of PR-10 proteins. Furthermore, this is a possible indicator of the location of epitopes binding to IgE; a fact that may hinder the application of *TcPR-10* gene in cocoa breeding.

The determination of three-dimensional structures of allergenic proteins allows to identify amino acids exposed on the molecular surface and that can act as IgE-binding epitopes [20]. Studies of structure analysis suggest the presence of common regions between Bet v 1, allergens, pollens, food, and PR proteins, therefore allowing the use of molecular modeling to predict protein structures. Using the SWISS-MODEL, the secondary structure of the protein TcPR-10 (Figure 2A) indicates a pattern similar to Bet v 1 and Pru av 1, differing by the presence of six β-sheets in TcPR-10 [12], [44]. The TcPR-10 protein has high homology with pollen and food allergens showing possible IgE-binding epitopes (Table 1) - as Bet v 1 and Pru av 1, respectively - for those parameters, two questions are of great importance respecting the parameters analyzed: 1) Is TcPR10 capable of triggering IgE-dependent hypersensitivity? Is there a possible expression of a mutant from the TcPR10 protein that maintains antifungal and ribonuclease activities and reduces or eliminates the potential for IgE production? The modeling of TcPR-10 protein has allowed to select Thr10, Ile30 and His45 sites for insertion of mutations. The amino acid Thr10 in TcPR-10 belongs to the β1 chain (3–12) located on the exposed surface of the protein (Figure 2A and C) is not situated near the conserved areas identified via SDAP. In turn, the amino acid Ile30 located in loop L2 (27–39) is situated in the cavity of the protein and near the conserved region 25DSDNLI30. The T10P and I30V substitutions inserted in TcPR-10 have been demonstrated in other PR-10 proteins - as an example, Bet v 1 [45] e Mal d 1 [22] - and are involved in the formation of IgE epitopes. The substitution of histidine for serine at position 45, located on β2 (40–46), was introduced for it precedes the conserved region of P-loop 47GDGGVG52 from TcPR-10. In Bet v 1, the residue from glutamic acid (E) at position 45 is considered as crucial for the recognition of antibodies [20], [21].

Analysis of the influence of site-directed mutations regarding the IgE binding capacity has demonstrated that the P-loop is not always involved in IgE binding and that similar amino acid substitutions in proteins with high identity not always result in loss of IgE binding capacity [22], [23], [46], [47]. In Mal d 1, a single point mutation at position 111 replacing serine for proline was responsible for reducing allergenicity [48]. The ability of TcPR-10 wt and TcPR-10 mut proteins to modulate the production response of IgE was examined in a urine model. The elevation of serum IgE levels in animals subjected to systemic sensitization by subcutaneous injection followed by exposure of the airways to the wild TcPR-10 indicate that this protein can modulate the response of total IgE. In line with the high levels of IgE antibodies, the number of leukocytes present in the BAL fluid was also higher in the wild-type TcPR-10 experimental group (Figures 6A and B), thus indicating the potential of TcPR10 in recruiting inflammatory cells, especially polymorph nuclear cells (Figure 6D). Histological analysis of lung tissue has confirmed the presence of cellular infiltration detected in BAL fluid samples (Figure 6D). On the other hand, there was a more attenuated inflammatory response in mice challenged with the TcPR-10 mutant protein characterized by lower levels of IgE and total leukocytes present in BAL fluid (Figure 6E).

Interestingly, the insertion of mutations has not altered the characteristics of ribonuclease and antifungal activity of the TcPR-10 protein, which are crucial in the resistance of *T. cacao*; hence, the method of insertion is of interest in the genetic improvement of these plants. In AhPR10, isolated protein from *Arachis hypogaea*, mutations in the K54N residue of the P-loop region has led to complete loss of ribonuclease activity, while other points of substitutions such as F148S and H150Q have partially affected the catalytic activity, thus indicating that these residues are important for the RNase function of this protein [19]. The maintenance of catalytic activity in mutant TcPR-10 (Figure 4) suggests that the amino acids Thr10, Ile30 and His45 may not determine this function in protein, but influences the allergenic character, as observed in the modulation of IgE production and recruitment of inflammatory cells. Some authors suggest that ribonuclease is associated with the capacity to inhibit fungal growth [19], [49]. The TcPR-10 mutant protein shows a small reduction in the time of degradation of RNA and also presents a reduction in the ability to inhibit fungal growth at a concentration of 10 µg/µl, as compared with the wild-type TcPR-10, thus indicating that these activities could be correlated. The presence of P-loop is also associated with the ribonuclease of PR-10 proteins. Site-directed mutations in three conserved amino acids (E95A, E147A, Y150A) and the construction of deletions in the P-loop protein SPE-16 isolated from *Pachyrrhizus erosus* seeds and classified as a member of the family PR-10 has shown different changes in ribonuclease activity [50]. In TcPR-10, the presence of conserved amino acid residues E97, E149 and Y151 is associated with ribonuclease activity [24].

The constitutive expression of genes in transgenic plants is the major concern about the allergenic potential of PR proteins. Sowoboda et al (1995) [51] emphasize that the conditions of pathogenic infections and stress can increase the level of expression of proteins homologous to Bet v 1 in pollens and other plant parts and thereby contribute to a significant increase in the incidence of type 1 hypersensitivity detected over the last years in industrialized countries. The overexpression of the *TcPR-10* gene in cocoa fruit should not be a critical factor limiting the genetic improvement of this species by transgenic techniques, since the processing of cocoa beans for chocolate production could reduce the possible allergenic activity of this protein. In addition, there are few reports of cases of clinical sensitivity to chocolate, what can be explained by the changes that proteins undergo as a result of the processing of cocoa beans [52]. Yet, the introduction of new forms of chocolate that are less processed, such as pieces of raw or toasted cocoa bean is a worrying concern, considering that the allergenicity and the importance of cross-reactivity of these products with other food allergens is unknown [53]. The possibility of a constitutive expression of the TcPR-10 protein in transgenic plants can become a public health problem, since the protein is expressed in pollen grains and could become a potential source of allergens triggering airway hypersensitivity, or the protein could show cross-reactivity with food allergens.

The experimental evidences generated from this study pave the way for the continuous use of the TcPR-10 protein as a potential antifungal agent that can be used in cocoa breeding through molecular engineering techniques. Besides maintaining the ribonuclease activity, the mutant TcPR-10 protein possibly modulates the immune response, showing characteristics that can be exploited in view of future applications in immunotherapies.

## Materials and Methods

### TcPR-10 Sequence

The *TcPR-10* gene (accession number ES439858) used in this study was identified from a cDNA library of cacao (cv. Catongo) inoculated by *M. perniciosa* [54]. Open reading frame (ORF) analysis of the nucleotide sequence obtained from the interaction cDNA library followed by homology search on BLAST [30] against sequences in the National Center for Biotechnology Information database identified a putative *PR-10* gene (insert size of 779 bp) with an ORF of 480 nucleotides encoding a protein of 159 amino acid residues. The *TcPR-10* gene was cloned into the pET28a (*Novagen*) expression vector as previously described [24].

### Identification of Potential Allergenic TcPR-10 Proteins through Bioinformatics Analysis

The analysis of the potential allergenicity of the amino acid sequence of the TcPR-10 protein was done using Bioinformatics tools available in Structural Database of Allergenic Proteins (SDAP, http://fermi.utmb.edu/SDAP). The sequence of TcPR-10 underwent alignments based on identity more than 35% in the window of 80 amino acids and identity of six contiguous amino acids of the TcPR-10 protein in a known allergen available in Base SDAP. The similarity of TcPR-10 with sequence available in the SDAP allergens was done using the statistical E-value and the property distance index PD. A low E-value (e.g. less than 10^−6^) indicates a high significance of the sequence match. The index PD values measure the similarity of two peptides based on five amino acid descriptors E1–E5 that were determined by a multidimensional scaling of 237 physicochemical properties of amino acids [55].



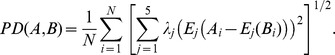
Where: λ*_j_* is the eigenvalue of the *j*-th *E* component, *E_j_*(*A_i_*) is the *E_j_* value for the amino acid in the *i*-th position from sequence *A*, and *E_j_*(*B_i_*) is the *E_j_* value for the amino acid in the *i*-th position from sequence *B.*

### Molecular Modeling

Comparative protein modeling was used to predict a structural model of TcPR-10 protein implemented by Swiss Pdb-Viewer v.3.7 accessible via the Expasy web Server (http://swissmodel.expasy.org/) [31], [56]. The homology modeling of protein TcPR-10 was performed using as a template the three-dimensional structure of the major cherry allergen, Pru av 1 (pdb:1e09_A), in solution, resolved by heteronuclear multidimensional NMR spectroscopy [17]. The modeling procedure started with the choice of the template based on the alignment of the sequences of proteins TcPR-10 wild and mutant to be modeled using the PSI-BLAST [30] with known three-dimensional (3-D) proteins structures available in the Protein Data BankProtein (pdb) (http://www.pdb.org). After align primary target sequence with template was submitted to modeling request to the Swiss Model Server.

Validation of the secondary structure was performed using PsiPred (Protein struture prediction Server - http://bioinf.cs.ucl.ac.uk/psipred/) [57]. The stereochemical quality of structural models of TcPR-10 wild type and mutant proteins obtained by homology modeling with the protein structure Pru av 1 (SolutionNMR) was assessed using the programs PROCHECK 3.4 [58] and ANOLEA (Atomic Non-Local Environment Assessment) [59]. The Root Mean Square Deviation (RMSD) differences from ideal geometries for bond lengths and bond angles were calculated on Pymol 3.0.

### Site-directed Mutagenesis

TcPR-10 inserted into the pET28a expression vector was modified by site-directed mutagenesis by overlap-extension based on polymerase chain reaction (PCR). Three oligonucleotide primer sets were constructed by site directed mutagenesis (Thr10Pro, Ile30Val, His45Ser) (Table 3). The PCR amplification was done at an annealing temperature of 50°C for 40 cycles of polymerization using *Taq* polymerase (Taq High Fidelity-*Fermentas*). The PCR product were digested with *Nde*I and *Sal*I and subcloned in expression vector pET28a (Novagen). The plasmid pET28a with TcPR-10 insert were transformed into *Escherichia coli* BL21(DE3) for protein expression. In the following TcPR-10 inserted into the pET28a without modification will be referred to as TcPR-10 wild type (TcPR-10 wt) and engineered mutant as TcPR-10 mutant (TcPR-10 mut).

**Table 3:** Primer sets used for mutation at positions Thr10Pro, Ile30Val and His45Ser in the *TcPR-10* gene.

Primers		Sequence (5′ to 3′)	Nucleotides
T10P	Forward	CAA GAG TTC **C** CC TGC TCA GTT G	22–43
	Reverse	C AAC TGA GCA GG**G** GAA CTC TTG	
I30V	Forward	C GAC AAC CTT **G** TC CCC AAA CTC	81–102
	Reverse	GAG TTT GGG GA**C** AAG GTT GTC G	
H45S	Forward	G GAG TTG ATT **AG** T GGA GAT GG	126–146
	Reverse	CC ATC TCC A**CT** AAT CAC CTC C	

The mutated codon is underlined and nucleotide substitution in bold.

**Figure 7:**
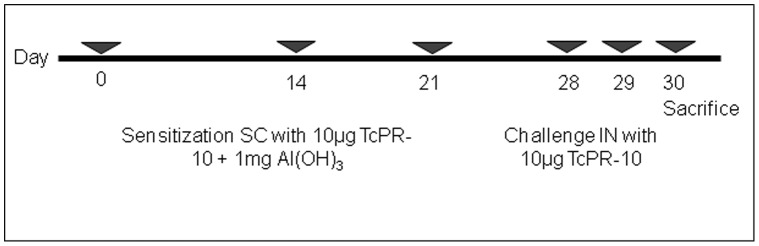
Experimental design of sensitization and challenge with TcPR-10 wild types and mutant.

### Expression and Purification of Recombinants Wild and Mutant-type TcPR-10

The expression of recombinant proteins TcPR-10 wt and TcPR-10 mut was obtained by the induction of a colony transformant grown in LB medium (Luria-Bertani) containing Kanamycin (50 µg.mL^−1^) and Chloramphenicol (34 µg.mL^−1^) under constant shaking at 37°C until reaching an OD_600_ between 0.5 and 0.7. Expression of recombinant proteins was induced by 1 mM *Isopropyl-ß-<$>\raster="rg1"<$>-thio-galactoside* (IPTG) followed by 15 h incubation at 18°C. The cells were centrifuged (11000×*g*, 20 min, 4°C) and pellets suspended in lyses buffer (50 mM phosphate buffer [PB], 300 mM NaCl, 2% of Nonidet, lysozyme at 0.1 mg.mL^−1^, pH 7.4) and sonicated by 4 min (30 s pulse/min, 75% output) (*Gex Ultrasonic processor 130, 130 W*). Subsequently, after centrifuged (11000×*g*, 20 minutos, 4°C) the samples were purified with TALON® Metal Affinity Resins (*Clontech Laboratories*) according to the manufacturer’s instructions. Expression of recombinant proteins was induced and harvested as described previously [24]. Expression and purification of recombinants TcPR-10 wt and TcPR-10 mut were analyzed by 15% Sodium dodecyl sulfate polyacrylamide gel electrophoresis (SDS-PAGE). Protein concentrations were determined by Bradford protein assay [60].

### Ribonuclease and Antifungal Activity of Mutant-type TcPR-10

Ribonuclease and antifungal activity of TcPR-10 mut was verified according to Bantignies and associates (2000) [61] with suggested modifications by Pungartnik and associates (2009). The RNA from *M. perniciosa* (Cp 553CEPLAC), growing in complete medium CPD (2% glucose, 2% peptone, 2% agar added for solid media), was extracted using phenol–chloroform method followed by ethanol precipitation and analyzed by 1% agarose gel electrophoresis. One microgram of RNA was incubated with TcPR-10 mutant protein (1 µg) at 25°C at different times (10 min, 20 min, 1 h, 2 h and 3 h). Positive control used wild-type TcPR-10 previously characterized with ribonuclease activity. Ribonuclease activity was determined by degradation total of RNA observed in 1% agarose gel. Antifungal activities of TcPR-10 mut protein were determined by inhibiting growth of dikaryotic *M. perniciosa* broken hyphae in increasing concentrations of the recombinant protein (0, 4, 8 e 10 µg/plate) [62].

### Allergen Sensitization and Challenge with Wild and Mutant-type TcPR-10

Female BALB/c mice, 8–10 weeks old, obtained from the Centro de Bioterismo (CEBIO) do Instituto de Ciências Biológicas da Universidade Federal de Minas Gerais, Brasil (UFMG) were used in this experiment. The animals were kept under conventionally standard housed conditions of the Instituto de Ciências Biológicas (ICB) of UFMG. This study, under the supervision of Dr. Abelmon da Silva Gesteira, was specifically approved by the Ethics Committee in Animal Experimentation (CEUA) of the Universidade Estadual de Santa Cruz (UESC) under protocol n. 001/09.

BALB/c mice were sensitized via subcutaneous (SC) in three time periods 0, 14 and 28 days, with the proteins and 1 mg Al(OH)3. For induction of allergic airway inflammation mice were challenged intranasal (IN) at 35 and 36 days as shown in Figure 7. The experiments were carried out in three experimental groups (n = 5): (i) 10 µg of TcPR10 wild protein [0.8 µg/µL], (ii) 10 µg of mutant [0.8 µg/µL] and (iii) control (PB, 300 mM NaCl).

### Measurement of Total IgE

Mice were bled after 24 h of the last IN challenge to antibody detection IgE by Enzyme-Linked Immunosorbent Assay (ELISA). Blood samples were centrifuged (5000×*g*, 10 min, 4°C) and serum was collected. Plates were coated with a rat anti-mouse IgE antibodies (UNLB) diluted in Coating Buffer (pH 9.6) (1∶250). Plates were incubated overnight in cold chamber at 4°C. Afterwards they were washed with PBS. The coated wells were blocked with PBS-Casein (50 mM phosphate buffer pH 7.2 with 0.25% of casein) for 1 h at room temperature. Detection of IgE was carried out using biotinylated rat antimouse IgE (1∶500) incubated for 1 h at room temperature. Streptavidin-peroxidase (Sigma-Aldrich) was used as a second-step reagent, developed using H_2_O_2_ with Ortho-Phenylenediamine (OPD) solved in citrate buffer, pH 5 (4 mg de OPD com 2 µL de H_2_O_2_ a 30%). The reaction was blocked by adding sulfuric acid 2N. The reactions were read on microplates BioRad Model 450 at 492 nm.

### Bronchoalveolar Lavage and Pulmonary Histopathology

For the determination of cellular infiltration in the lung, bronchoalveolar lavage fluids, were collected. After the sacrifice, lungs were washed with 1 mL of cold PBS containing bovine serum albumin (0.03%). Total number of cells was determined by counting in a Neubauer chamber. The count was done by two independent investigators under the optic microscope (OLYMPUS B12).

After the collection of the bronchoalveolar lavage the lungs were removed and fixed with 10% buffered formaldehyde (v/v) (0.1 M phosphate buffer, pH 7.4) and processed in a standard manner. For the histological examination, fixed embedded lungs were sectioned into 5 µm sections, deparaffinized with xylene and graded ethanol and stained with haematoxylin and eosin (H&E). For the morphological evaluation of lung tissues the preparations were carried out microscopically (OLYMPUS B12) and images captured in digital camera for evaluation of the symptons of the airway inflammation as expression of infiltration of inflammatory cells.

### Statistical Analysis

Data were subjected to the Shapiro Wilk normality test followed by analysis of variance for parametric data with Tukey Test (α = 0.05) to compare the means of experimental groups with control. For nonparametric data the Kruskal-Wallis test was used. Data are shown as mean (M) and standard deviation (SD). Differences were considered significant for *p* values <0,05. Analyses were carried out using the Bioestat v4.0 [63] and GraphPad PRISM v.4.0 [64].
